# Exploring the Relationship of Perivascular Adipose Tissue Inflammation and the Development of Vascular Pathologies

**DOI:** 10.1155/2022/2734321

**Published:** 2022-02-08

**Authors:** Afifah Zahirah Abd Rami, Adila A. Hamid, Nur Najmi Mohamad Anuar, Amilia Aminuddin, Azizah Ugusman

**Affiliations:** ^1^Department of Physiology, Faculty of Medicine, Universiti Kebangsaan Malaysia, Jalan Yaacob Latif, Cheras, 56000 Kuala Lumpur, Malaysia; ^2^Center for Toxicology & Health Risk Studies, Faculty of Health Sciences, Universiti Kebangsaan Malaysia, Jalan Raja Muda Abd Aziz, 50300 Kuala Lumpur, Malaysia

## Abstract

Initially thought to only provide mechanical support for the underlying blood vessels, perivascular adipose tissue (PVAT) has now emerged as a regulator of vascular function. A healthy PVAT exerts anticontractile and anti-inflammatory actions on the underlying vasculature via the release of adipocytokines such as adiponectin, nitric oxide, and omentin. However, dysfunctional PVAT produces more proinflammatory adipocytokines such as leptin, resistin, interleukin- (IL-) 6, IL-1*β*, and tumor necrosis factor-alpha, thus inducing an inflammatory response that contributes to the pathogenesis of vascular diseases. In this review, current knowledge on the role of PVAT inflammation in the development of vascular pathologies such as atherosclerosis and hypertension was discussed.

## 1. Introduction

Cardiovascular diseases (CVD) have been widely known for decades as the leading cause of mortality worldwide. According to the World Health Organization (WHO), in 2019, an estimated 17.9 million deaths due to CVD were recorded, representing 32% of global deaths [1]. This alarming situation raises the importance of investigating the pathophysiological aspects of CVD. Perivascular adipose tissue (PVAT) surrounds most blood vessels and has been implicated in the pathophysiology of CVD due to its proximity and crosstalk with the underlying vasculature, with PVAT inflammation suggested to be contributing to the development of vascular diseases. A link between PVAT, inflammation, and CVD was first discovered two decades ago when a study showed an increase in leukocyte infiltration in the PVAT in response to coronary angioplasty [2]. This signifies the role of PVAT inflammation in the pathophysiology of CVD and as a potential future therapeutic target.

## 2. Perivascular Adipose Tissue

PVAT consists of adipocytes that surround most systemic blood vessels, except the cerebral vasculature [3]. Structurally, PVAT consists of adipocytes, fibroblasts, stem cells, lymphocytes, and macrophages. The characteristics of PVAT differ in different anatomical sites. For example, thoracic aorta PVAT (tPVAT) and abdominal aorta PVAT (aPVAT) are two subtypes of PVAT which possess different phenotypes and functions. In rodents, tPVAT's characteristics are more similar to brown adipose tissue (BAT), while aPVAT is phenotypically a mixture of white adipose tissue (WAT) and BAT, whereas in humans, coronary PVAT's characteristics are more similar to WAT [4–6]. tPVAT is mainly involved in lipolysis and heat generation and facilitates vascular relaxation via adipokine release, whereas aPVAT is involved in lipid storage and cytokine secretion and contains more macrophages and immune cells [7]. Hence, aPVAT is regarded to be more prone to proinflammatory activity and is proatherogenic compared to tPVAT.

Studies have shown that each type of adipocyte is derived from a specific precursor and is differentiated at separate times during embryogenesis [8, 9]. Most white adipocytes are differentiated from Myf5^+^ and PAX3^+^ precursors or Myf5^−^/Pax3^+^, while brown adipocytes are derived from paraxial mesoderm Myf5^+^/Pax3^+^/Pax7^+^/En1^+^, a common precursor of myocytes [10]. Peroxisome proliferator-activated receptor- (PPAR-) *γ* is a transcription factor that is involved in the regulation of gene expression and differentiation of adipocytes [11]. Deletion of PPAR-*γ* during BAT adipogenesis impairs PVAT development and increases local inflammation, which often leads to the progression of atheromatous plaque and myocardial injury in vivo [12, 13]. The activation of PPAR-*γ* has been shown to attenuate arterial stiffening and reduce inflammatory and oxidative stress in the PVAT of obese mice [14]. These findings highlight the significant role of PPAR-*γ* in PVAT in modulating inflammation and risk of vascular diseases.

**Figure 1:**
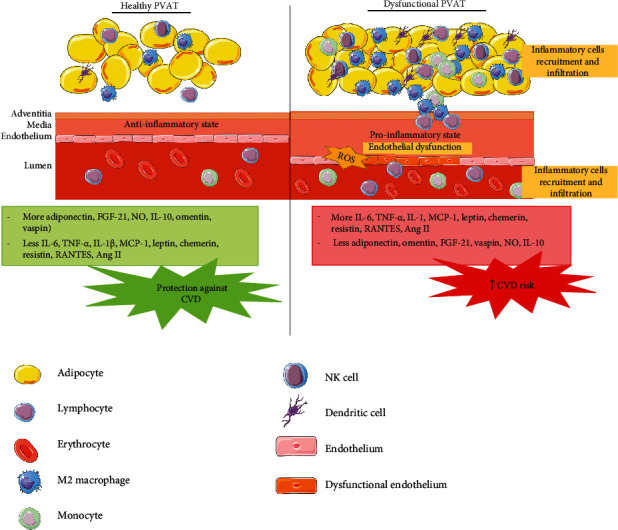
The anti-inflammatory state of healthy perivascular adipose tissue (PVAT) compared to the proinflammatory state of dysfunctional PVAT. CVD: cardiovascular diseases; FGF-21: fibroblast growth factor-21; IL: interleukin; MCP-1: monocyte chemoattractant protein-1; NO: nitric oxide; RANTES: regulated on activation, normal T cell expressed, and secreted; ROS: reactive oxygen species; TNF-*α*: tumor necrosis factor-alpha.

Although PVAT was initially regarded as a structural support organ for the vasculature, recent findings have demonstrated its physiological importance, especially in regulating vascular tone and function [15]. In healthy subjects, PVAT functions as an endocrine and paracrine organ that secrets more anti-inflammatory, antiatherogenic, and vasorelaxant adipokines such as adiponectin, omentin, vaspin, angiotensin 1-7, methyl palmitate, and nitric oxide (NO), which contribute to its anticontractile, anti-inflammatory, and antiatherogenic actions [16, 17]. However, in the presence of vascular diseases such as hypertension, atherosclerosis, and obesity, PVAT becomes dysfunctional. A dysfunctional PVAT secretes less anti-inflammatory adipocytokines and more proinflammatory adipocytokines such as leptin, interleukin- (IL-) 6, tumor necrosis factor-alpha (TNF-*α*), and monocyte chemoattractant protein-1 (MCP-1) [18, 19]. These trigger inflammatory responses that lead to vascular dysfunction and an increased risk of developing CVD (Figure 1).

## 3. PVAT-Derived Anti-Inflammatory Adipocytokines

Adiponectin is one of the most abundant adipokines secreted by PVAT with anticontractile and anti-inflammatory effects on the vascular wall. Adiponectin acts via two types of receptors: adiponectin receptor 1 (AdipoR1) and adiponectin receptor 2 (AdipoR2) [20]. Adiponectin exerts its anti-inflammatory effects by decreasing the expression of proinflammatory cytokines such as IL-6 and TNF-*α* and suppressing the production of cellular adhesion molecules by inhibiting the nuclear factor kappa-B (NF-*κ*B) pathway [21]. Moreover, hypoadiponectinemia results in endothelial dysfunction, and this is mediated by Nod-like receptor family pyrin domain-containing 3 (NLRP3) inflammasome activation [22]. Adiponectin knock-out mice showed an increase in the gene expression of inflammatory markers such as TNF-*α* and MCP-1. This further proves the anti-inflammatory action of adiponectin [23].

Omentin secreted by PVAT also has an anti-inflammatory effect. In obese mice, omentin reduces the expression of proinflammatory cytokines such as IL-6, IL-1*β*, and TNF-*α* and increases the secretion of other anti-inflammatory adipocytokines such as adiponectin and IL-10 through the inhibition of thioredoxin-interacting protein (TXNIP)/NLTP3 signaling pathway [24]. Furthermore, omentin reduces oxidative stress, mitochondrial dysfunction, proinflammatory cytokines (IL-6, IL-8, and MCP-1), cyclooxygenase-2 (COX), and prostaglandin E2 (PGE2) in lipopolysaccharide-induced macrophages [25]. Omentin protects against vascular endothelial dysfunction by suppressing endoplasmic reticulum (ER) and oxidative stress. This is achieved through the activation of AMP-activated protein kinase (AMPK)/PPAR-*δ* pathway that stimulates NO release [26]. In free fatty acid-induced endothelial cells, omentin decreases proinflammatory agents (MCP-1, IL-6, IL-1, ICAM-1, TNF-*α*) and NF-*κ*B activation [27].

Fibroblast growth factor-21 (FGF-21) is a growth factor expressed in multiple tissues and organs such as adipose tissue, liver, and pancreas that regulates insulin signaling, glucose, and lipid metabolism [28]. FGF-21 exerts anti-inflammatory effects in macrophages and obese adipose tissue via various inflammatory signaling pathways [29, 30]. Treatment of apolipoprotein E-deficient (ApoE^−/−^) mice with FGF-21 reduced atherosclerosis formation by increasing adiponectin expression and inhibiting ER stress, NLRP3 inflammasome activation, and factor-associated suicide (FAS) signaling [31–33]. FGF-21 also improves oxidative-stress-induced endothelial dysfunction by activating the calcium/calmodulin-dependent protein kinase kinase 2 (CaMKK2)/AMPK*α* pathway [34].

Vaspin, which is also known as visceral adipose tissue-derived serine protease inhibitor, is an anti-inflammatory adipokine that improves insulin sensitivity [35]. A study has reported that vaspin inhibits the gene expression of leptin receptor, production of TNF-*α*, and NF-*κ*B activation in leptin-induced rat chondrocytes [36] This suggests an important role of vaspin produced by PVAT in modulating inflammatory reactions. Meanwhile, interleukin-10, which is produced by T cells, B cells, and macrophages in the PVAT, plays a significant role in preventing inflammation [37]. IL-10 suppresses proinflammatory cytokine secretion and prevents macrophage and dendritic cell maturation [38]. IL-10 acts on heterodimeric IL-10 receptors (IL-10R1, IL-10R2), which activates the Janus kinase (JAK)/STAT signaling pathway. This leads to inhibition of proinflammatory mediator production [39].

Another PVAT-derived anti-inflammatory factor is NO. Vascular NO is produced mainly by endothelial NO synthase (eNOS) [40]. NO inhibits vascular smooth muscle proliferation and migration, platelet aggregation, leukocyte adhesion, and inflammation [41]. eNOS, which is mainly expressed in the endothelium, has also been found to be present in PVAT [42]. PVAT-derived adiponectin increases eNOS phosphorylation, thus enhancing NO production. Endothelial dysfunction is characterized by the reduction of NO bioavailability, and this is associated with inflammation and development of atherosclerosis and hypertension [42].

## 4. PVAT-Derived Proinflammatory Adpocytokines

IL-6 is one of the most studied proinflammatory cytokines secreted by PVAT. IL-6 can directly act on endothelial cells to increase superoxide production, thus leading to endothelial dysfunction [18]. Another prominent cytokine that modulates PVAT inflammatory response is TNF-*α*. TNF-*α* is released by a few types of cells including monocytes, vascular cells, and adipocytes. It inhibits the eNOS expression and stimulates the production of reactive oxygen species (ROS) via activation of the NF-*κ*B pathway [18]. Other proinflammatory cytokines released by PVAT include MCP-1 and IL-1*β*. MCP-1 plays a crucial role in facilitating the infiltration of macrophages into the vascular wall and is often associated with the pathogenesis of atherosclerosis [43]. Meanwhile, IL-1*β* induces the MCP-1 expression via NF-*κ*B and activator protein-1 (AP-1) activation [44].

Chemerin, also known as tazarotene-induced gene 2 (TIG2), is a chemoattractant protein that regulates the immune response, metabolism, and inflammation. Although some studies have associated chemerin with anti-inflammatory actions, its function is mainly considered as proinflammatory [45]. Chemerin is expressed in adipose tissue including PVAT and visceral adipose tissue [46, 47]. Both chemerin and its receptor, CMKLR1, are upregulated in obesity [48]. Furthermore, chemerin recruits dendritic cells into adipose tissue, thus increasing the inflammatory reaction in adipocytes [49]. Chemerin also stimulates the recruitment and retention of macrophages at inflammation sites by inducing macrophage adhesion to extracellular matrix proteins and adhesion molecules [50]. The level of chemerin is closely correlated with the level of other proinflammatory cytokines such as TNF-*α*, C-reactive protein (CRP), and IL-6, which further supports chemerin's proinflammatory action [51].

Leptin has been suggested as a PVAT-derived proinflammatory factor as its expression can be stimulated by other proinflammatory mediators such as IL-1 and TNF-*α*, and its concentration increases during pathological conditions such as fever and sepsis [52]. Leptin activates monocytes, leukocytes, and macrophages to secrete TNF-*α*, IL-6, and IL-12, increases CC-chemokine ligand production in macrophages, and stimulates ROS generation [38]. Additionally, leptin causes endothelial dysfunction [53] by inducing CRP production, cellular adhesion molecules, and platelet tissue factors in endothelial cells [54].

Resistin is another proinflammatory adipokine released by PVAT that enhances the expression of IL-6, IL-1*β*, and TNF-*α* via NF-*κ*B signaling [55]. Other than adipocytes, resistin is released by immune cells including monocytes and macrophages, and this suggests its role in atherogenesis. Moreover, resistin causes endothelial dysfunction by enhancing proinflammatory markers such as MCP-1, TNF-*α*, IL-6, long pentraxin 3, IL-1*β*, vascular cell adhesion molecule-1 (VCAM-1), and intercellular adhesion molecule-1 (ICAM-1). Besides, resistin promotes oxidative stress and ER stress, thus leading to mitochondrial dysfunction and redox imbalance [56, 57].

The components of the renin angiotensin aldosterone system are expressed in PVAT. Its bioactive peptide, angiotensin II (Ang II), exerts its inflammatory effect by stimulating the expression of adhesion molecules and cytokines such as MCP-1 and IL-6 [58]. Ang II is also reported to induce immune cells including T lymphocytes, M1 and M2 macrophages, and dendritic cell infiltration in PVAT [59, 60]. Interestingly, a large proportion of these cells, particularly T lymphocytes, bear regulated on activation, normal T cell expressed, and secreted (RANTES) CC chemokine receptor (CCR)1, CCR3, and CCR5 receptors. Expression of RANTES chemokine receptors is stimulated by Ang II [61]. RANTES, also known as CC chemokine ligand 5 (CCL5), is present in both human and mouse adipose tissue. RANTES acts as a chemoattractant for inflammatory cells, particularly T cells, and it is associated with the development of atherosclerosis and hypertension [62]. The summary of anti- and proinflammatory adipocytokines secreted by PVAT and its involvement in vascular diseases is summarized in Table 1.

## 5. PVAT Inflammation in Vascular Diseases

### 5.1. Atherosclerosis

Atherosclerosis is an inflammatory disease involving the formation of fibrofatty plaques in the arterial wall that can progress to several chronic diseases such as coronary artery disease (CAD), stroke, and peripheral artery disease [63]. The atherosclerotic plaque consists of the accumulation of fatty substances, cholesterol, calcium, cellular waste products, and fibrin [64]. Several risk factors are closely related to atherosclerosis, including hypertension, tobacco smoking, obesity, and diabetes [65]. Traditionally, the pathogenesis of atherosclerosis has been described as an “inside-to-outside” model that starts with endothelial dysfunction, inflammation, and formation of foam cells [66]. However, in most vascular studies, PVAT was removed from the underlying blood vessels prior to experiments as PVAT was considered to be a nonvascular and inactive tissue [67].

Interestingly, more current studies propose that the crosstalk between PVAT and the underlying vasculature happens in two directions, with an “outside-to-inside” inflammatory signaling activated by the dysfunctional PVAT [68–70]. For example, it was demonstrated that inflammation in the PVAT and adventitial layer happened prior to the development of endothelial dysfunction and formation of atherosclerotic plaques in apolipoprotein E-deficient (ApoE^−/−^) mice [71]. Perivascular adipocytes send signals to both immune cells and endothelial cells via the release of adipocytokines to modulate the inflammatory crosstalk in atherogenesis [72]. PVAT plays a crucial role in the pathogenesis of atherosclerosis through mechanisms involving endothelial dysfunction and inflammatory cell recruitment and infiltration [70, 73].

Immune cell infiltration is an important step in PVAT inflammation and atherosclerosis. T cell infiltration in PVAT may occur prior to macrophage infiltration in mice [74]. Both proatherogenic (CD4^+^ T helper (Th), CD8^+^ T cytotoxic (Tc)), and atheroprotective (T regulatory (Treg)) T cells are found in PVAT [75–77]. Th1, Tc1, and Th17 cells release proinflammatory cytokines such as interferon-*γ* (IFN-*γ*), IL-7, and TNF-*α*, whereas Treg cells secrete anti-inflammatory cytokine, IL-10 [78, 79]. Subpopulation of T cells such as natural killer T cells also release IFN-*γ* and TNF-*α* in PVAT [80].

### 5.2. Mechanisms Linking PVAT Inflammation to Atherosclerosis

The endothelium acts as a physical barrier between blood and the vascular wall. Besides, the endothelium also secretes bioactive molecules involved in the regulation of vascular tone, vascular remodeling, inflammatory process, and thrombosis [81]. NO produced by eNOS is an important antiatherogenic molecule, and reduced NO bioavailability is the hallmark of endothelial dysfunction [82]. Endothelial dysfunction is well established as the precursor of atherosclerosis. A dysfunctional endothelium loses its physiological characteristics and transforms into a proinflammatory, prothrombotic, and vasoconstrictor state, thus promoting atherosclerosis [83].

As described earlier, in physiological condition, PVAT produces a range of vasoprotective adipocytokines with antiatherogenic effects such as adiponectin, NO, and hydrogen sulfide (H_2_S) [84]. Reduced NO and H_2_S have been shown to worsen atherosclerosis progression [82, 85]. Unsurprisingly, since atherosclerosis is closely related to obesity, PVAT-derived NO, H_2_S, and adiponectin levels were reduced in obese animals [86–89]. It is postulated that in obesity, increased PVAT mass and hyperthrophic adipocytes in PVAT promote endothelial dysfunction and atherosclerosis through increased oxidative stress and inflammation [90, 91].

Breakdown of fat in hypertrophic PVAT releases free fatty acids (FFA) into the vasculature. FFA causes phosphorylation of insulin receptor substrate 1 (IRS-1) present in PVAT and other vascular cells by activating NF-*κ*B, protein kinase C (PKC), and Toll-like receptors [92]. This decreases the activation of downstream PI3K/Akt signaling, leading to inhibition of eNOS expression and NO synthesis [93]. Besides, PKC activation by FFA causes eNOS coupling, resulting in further reduction in NO synthesis and production of reactive oxygen species (ROS) [7, 94]. Furthermore, PKC induces the synthesis of endothelin-1 (ET-1), which is a potent vasoconstrictor. Reduced NO bioavailability, increased vasoconstrictor, and ROS accumulation result in endothelial dysfunction and enhance the development of atherosclerosis [92].

Subsequently, monocyte recruitment and activation ensue. Dysfunctional PVAT releases more pro-inflammatory adipocytokines such as TNF-*α*, leptin, and IL-6 that induce the expression of cellular adhesion molecules like VCAM-1 and ICAM-1 on the endothelial cells. Cellular adhesion molecules promote the adherence and migration of monocytes into the subendothelial layer [95–97]. The migrated monocytes transformed into macrophages that secrete proinflammatory cytokines such as MCP-1, TNF-*α*, IFN-*γ*, and IL-6, which further aggravate monocyte recruitment and low-density lipoprotein (LDL) oxidation [98].

The macrophages engulf oxidized LDL (oxLDL) through scavenger receptors including leptin-like oxLDL receptor-1 and CD36, leading to formation of foam cells. Foam cells are the hallmark of initial stage of atherosclerosis [98]. Furthermore, PVAT-derived adipocytokines such as leptin, TNF-*α*, visfatin, and IL-6 stimulate vascular smooth muscle cell (VSMC) proliferation and migration, which is an important step in neointima formation [99, 100]. Recent evidence demonstrates that in animal models, infiltration of macrophages in the PVAT and adventitial layer is more marked compared to the intimal layer [101, 102]. This further supports the notion that PVAT inflammation plays a significant role in atherosclerosis development.

Proinflammatory chemokines such as macrophage inflammatory protein 1-*α* (MIP-1*α* or CCL3), MCP-1, and RANTES attract immune cells to the site of perivascular inflammation in atherosclerotic ApoE^−/−^ mice [103–105]. PVAT of ApoE^−/−^ mice was also found to have increased levels of IL-6 and IL-1 as well as macrophages and T cell infiltration [106]. However, there was a decrease in the number of B-1 cells that secrete antiatherosclerotic IgM in ApoE^−/−^ mouse PVAT. This further worsens the atherosclerotic lesion formation in the mouse coronary artery and aorta [107]. Additionally, microRNA-19b in endothelial cell-derived microparticles promotes atherosclerosis progression in ApoE^−/−^ mice by increasing the secretion of proinflammatory cytokines (IL-6, IL-10, and TNF-*α*) and inducing macrophage infiltration in PVAT [108].

Interestingly, transplantation of normal PVAT from wild-type mice decreased the size of atherosclerotic plaque in ApoE^−/−^ mice. This effect was mediated by the anti-inflammatory action of transforming growth factor (TGF)-*β*1 [109]. Besides, adiponectin obtained from PVAT decreased carotid collar-induced atherosclerosis by stimulating macrophage autophagy [110]. A study demonstrated that xenotropic and polytropic retrovirus receptor 1 (Xpr1), a macrophage regulator, and TATA-box binding protein associated factor 3 (Taf3), a core transcription factor, were upregulated in the PVAT of ApoE^−/−^ mice. Furthermore, an upregulation of the Taf3 and Xpr1 expression was also detected in human atherosclerotic plaques [111]. This suggests that Taf3 and Xpr1 have a role in modulating the chronic inflammatory phenotype of PVAT.

Data from human studies demonstrated that PVAT derived from patients with CVD have increased expression of proinflammatory genes and decreased expression of anti-inflammatory adiponectin [112–114]. For instance, the epicardial adipose tissue of patients with coronary atherosclerosis showed increased expression of IL-1*β*, IL-6, and TNF-*α* [113] and lower expression of adiponectin [114]. The levels of inflammatory mediators such as IL-1*β*, IL-6, and IL-10 were elevated in the pericoronary PVAT of patients with CAD compared to patients without CAD [115]. This suggests the influence of PVAT inflammation on atherosclerosis development through an outside-to-inside manner.

Besides, the levels of protein inhibitor of activated STAT1 (PIAS1), a key negative regulator of inflammation, were reduced in PVAT obtained from patients with atherosclerotic vessel disease [116]. PIAS1 downregulates inflammation by inhibiting STAT1 and NF-*κ*B signaling pathways [117, 118]. Besides, the number of macrophages in the PVAT correlates with the number of immune cells in the atherosclerotic plaque [119–121].

**Figure 2:**
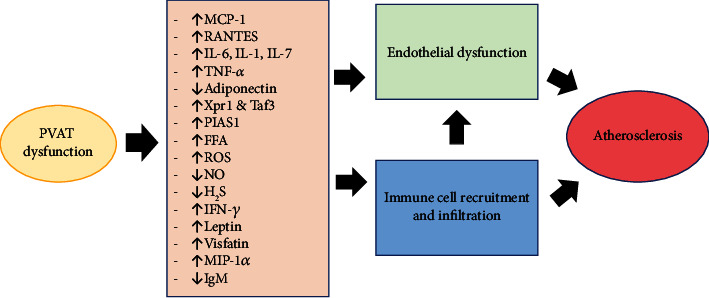
Pathways involving perivascular adipose tissue (PVAT) inflammation in atherosclerosis. FFA: free fatty acid; H_2_S: hydrogen sulfide; IFN-*γ*: interferon-*γ*; IL: interleukin; IgM: immunoglobulin M; MCP-1: monocyte chemoattractant protein-1; MIP-1*α*: macrophage inflammatory protein 1-*α*; NO: nitric oxide; PIAS1: protein inhibitor of activated STAT1; RANTES: regulated on activation, normal T cell expressed, and secreted; ROS: reactive oxygen species; Taf3: TATA-box binding protein associated factor 3; TNF-*α*: tumor necrosis factor-alpha; Xpr1: xenotropic and polytropic retrovirus receptor 1.

Unstable plaque is an important culprit for the occurrence of acute coronary syndrome. Apart from the established factors that influence plaque stability such as intraplaque neovascularization, inflammation, and intraplaque protease activity [122], PVAT inflammation has also been suggested to affect plaque stability. The number of macrophages was higher in PVAT near unstable plaques compared to the PVAT near stable plaques [123]. Furthermore, endoplasmic reticulum (ER) stress transforms adipose tissue to a proinflammatory phenotype [124]. ER stress in PVAT contributes to plaque instability by stimulating a proinflammatory factor, granulocyte macrophage colony-stimulating factor (GM-CSF), via NF-*κ*B activation [125]. GM-CSF causes adipose tissue inflammation by recruiting and activating M1 macrophages [126]. The mechanisms linking PVAT inflammation and atherosclerosis are summarized in Figure 2.

## 6. Hypertension

There are several mechanisms involving PVAT that contribute to hypertension, such as loss of PVAT anticontractile effect, increase in PVAT proinflammatory adipocytokines, decrease in PVAT anti-inflammatory adipocytokines, immune cell infiltration, activation of local RAAS, and increase in vascular oxidative stress [12]. The initial site of inflammation during the development of hypertension is in the PVAT and in the border between the PVAT and the adventitial layer [61, 127, 128].

### 6.1. Mechanisms Linking PVAT Inflammation to Hypertension

In hypertension, PVAT releases more proinflammatory adipocytokines such as IL-6, IL-17, IL-8, IL-23, IL-1*β*, TNF-*α*, and TGF-*β* [129] and less anti-inflammatory adipocytokines such as adiponectin, IL-10, and IL-4 [129, 130]. Consequently, there is infiltration of immune cells in the PVAT, loss of PVAT anti-contractile action, and increased vascular resistance [19]. These events are mediated by numerous inflammatory cells and cytokines. For example, complement C5a mediates the reduction in PVAT adiponectin release [131], RANTES mediates the invasion of lymphocyte T cells into the perivascular space [38], and IFN-*γ* is released by CD8^+^ cells that invade the PVAT [132]. All these changes intensify PVAT dysfunction and proinflammatory crosstalk between PVAT and the underlying hypertensive vessels.

A study involving spontaneously hypertensive mice induced by perilipin-1 deletion showed that the mice had higher aortic blood pressure, loss of PVAT anti-contractile effect, and decreased adiponectin expression. These findings are associated with increased expression of MCP-1, TNF-*α*, and IL-6 in the aorta [133]. Meanwhile, PVAT of DOCA-salt hypertensive mice displayed an increase in the complement C3 expression, leading to increased proinflammatory M1 macrophages and decreased anti-inflammatory M2 macrophage expression in the PVAT [134]. Recruitment of proinflammatory macrophages in the PVAT of DOCA-salt hypertensive mice enhances complement activation and promotes TNF-*α* release, thereby reducing adiponectin expression [135].

During the progression of hypertension, accumulation of immune cells has been reported in the PVAT surrounding both the aorta and mesenteric arteries of hypertensive animals [18]. Hypertension is associated with a significant increase in T cell infiltration in the PVAT. This promotes inflammation and endothelial disyfunction via NF-*κ*B-dependent, Notch ligand Jagged 1-regulated integrin, and adhesion molecule expression [136, 137]. Additionally, both CD4^+^ and CD8^+^ T cell subpopulations are increased in the PVAT of hypertensive mice. T cell- and monocyte-deficient mice exposed to various hypertensive stimuli showed reduced perivascular inflammatory reaction [75, 138].

Macrophage infiltration in PVAT is also significantly increased in hypertension, and this is regulated by T cell-dependent mechanisms [61]. Depriving the lymphocyte adaptor protein (Lnk) gene that encodes the negative regulator of T cell activation promotes PVAT inflammation, as the number of macrophages increase in both aorta and adipocytes [139]. The increase in blood pressure also corresponds with the expression of macrophage chemokine receptors CCR2 and its ligands such as CCL2, CCL7, CCL8, and CCL12 in the PVAT [140]. Moreover, the expression of proinflammatory M1 macrophage is increased in hypertension compared to healthy condition, whereby the anti-inflammatory M2 macrophage is more dominant [61].

Overactivation of RAAS is critical to the development of hypertension. Adipose tissue has been suggested as one of the main sites of RAAS activation in hypertensive patients [92]. In obese hypertensive patients, adipose tissues are the major source of RAAS [141]. All components of RAAS can be found in PVAT except renin [142, 143]. Angiotensinogen and Ang II levels in PVAT were significantly increased in SHR [144]. Knockout of angiotensinogen gene in PVAT successfully decreased local Ang II production in mice PVAT [145].

PVAT of Ang II-induced hypertensive mice displayed a higher number of immune cells such as macrophages, leukocytes, T cells, and dendritic cells. Ang II induction also enhances RANTES, MCP-1, and CCL3 expression in the periaortic PVAT and the aortic wall of the mice [61, 146]. Moreover, activation of angiotensin II type 1 receptor (AT1R) in PVAT promotes vascular inflammation and endothelial dysfunction [147].

Sirtuin-3 (SIRT3), a mitochondrial NAD^+^-dependent deacetylase that regulates multiple metabolic enzymes, plays a significant role in Ang II-related PVAT inflammation. It was reported that Ang II promotes PVAT inflammation and fibrosis by stimulating NLRP3/IL-I*β* pathway in myeloid SIRT3 knockout mice [148]. Activation of NLRP3 inflammasome is involved in vascular inflammation and its blockade has been proven to reduce adipose tissue inflammation and fibrosis (P. [149, 150]). SIRT3 is therefore a potential therapeutic target to inhibit NLRP3-related PVAT inflammation and fibrosis [148].

In addition to the direct actions of Ang II, aldosterone that is released in response to Ang II stimulation also has a proinflammatory action on PVAT [151]. Treatment with aldosterone receptor antagonists improves endothelial function, reduces oxidative stress, and decreases blood pressure [152, 153]. Meanwhile, treatment with angiotensin receptor blockers lower the release of Ang II from PVAT. This leads to the release of PVAT-derived relaxing factors that stimulate vasodilation through the opening of voltage-gated potassium channels in the vascular smooth muscle cells [147, 154, 155].

A complex reactive oxygen species (ROS) machinery containing NADPH oxidase (Nox) and antioxidative enzymes are also expressed in PVAT [156, 157]. Chronic oxidative stress enhances vascular inflammation in hypertension, with Nox being the main source of superoxide in the vasculature [158]. ROS derived from Nox in PVAT induces endothelial dysfunction by scavenging endothelial NO and modulating perivascular inflammation [90, 159].

SHR showed greater T cell accumulation in the PVAT and higher mRNA expression of Nox1 and Nox 4 in the vessels, an effect that was exacerbated with aging [160]. Mice with the overexpression of Nox p22phox catalytic subunit have enhanced vascular superoxide production and increased PVAT leukocyte infiltration that worsens hypertension progression [161]. Meanwhile, mice with loss of Nox subunit such as p47phox, Nox1, and Nox4 show protection against hypertension [162, 163]. Surprisingly, treatment of SHR with GKT137831, a dual inhibitor of Nox1/4, raised both blood pressure and PVAT macrophage infiltration and accelerated vascular aging. This observation was associated with increased expression of proinflammatory chemokine expression (CCL2 and CCL5) in the PVAT [160]. Therefore, these changes need to be considered when designing a therapy that targets Nox to treat hypertension.

Hypertension is more common in people who are obese compared to people who are lean [164]. Animal studies showed that obesity leads to increased PVAT mass and adipocyte hypertrophy with signs of PVAT inflammation, endothelial dysfunction, and altered release of adipocytokines. The release of proinflammatory cytokines such as TNF-*α*, MCP-1, IL-6, and IL-8 is markedly enhanced, whereas the anti-inflammatory adipokine, adiponectin, is markedly reduced [90, 91]. TNF-*α* inhibits adiponectin and NO production and stimulates ET-1 release. Imbalance between the vasoconstrictor ET-1 and the vasodilators such as NO and adiponectin is implicated in obesity-induced endothelial dysfunction and hypertension [42]. Adiponectin level is restored in hypertensive patients who received antihypertensive agents to control their blood pressure, which signifies the beneficial effect of adiponectin in hypertension [165].

**Figure 3:**
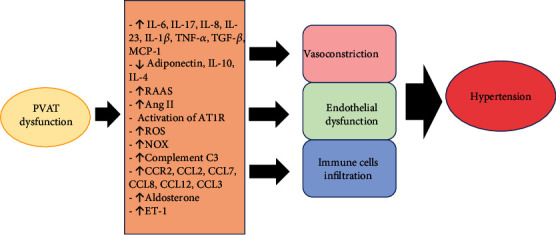
Pathways involving perivascular adipose tissue (PVAT) inflammation in hypertension. Ang II: angiotensin II; AT1R: angiotensin II type 1 receptor; C3: component 3; CCR: CC chemokine receptor; CCL: CC chemokine ligand; ET-1: endothelin-1; IL: interleukin; MCP-1: monocyte chemoattractant protein-1; NOX: NADPH oxidase; RAAS: renin-angiotensin-aldosterone system; ROS: reactive oxygen species.

PVAT inflammation and altered adipocytokine profile in obesity have significant effects on the anticontractile effect of PVAT and blood pressure [166]. Obesity causes the loss of anticontractile function of PVAT [90] and impairs endothelium-dependent vasorelaxation which contribute to hypertension [167]. The loss of anticontractile function of PVAT correlates with the increase in blood pressure in rodent models of diet-induced obesity [87]. Like obese animal models, the anticontractile effect of PVAT is lost in obese patients [168]. TNF-*α* expression is also elevated in the vascular wall and PVAT isolated from the small arteries of obese patients [42]. Six months following bariatric surgery, reduction in the body weight of obese patients is accompanied by improvement in their PVAT adipocytokine profile, PVAT anticontractile function, and blood pressure [168]. The mechanisms linking PVAT inflammation and hypertension are summarized in Figure 3.

## 7. Conclusion

PVAT inflammation plays a mechanistic role in the pathogenesis of vascular diseases such as atherosclerosis and hypertension (Table 2). The vicinity of PVAT as an active endocrine and paracrine organ that produces various adipocytokines as well as the related changes in this tissue supports the idea that alteration in PVAT phenotype contributes to disease processes in the adjacent vascular wall. Inflammation leads to PVAT dysfunction through the release of various proinflammatory adipocytokines and immune cell infiltration. Although the mechanisms on how PVAT inflammation is linked to vascular diseases are not entirely clear, hence the need for more mechanistic studies to explore this matter, the available evidence shows that PVAT inflammation occurs at the initial part of vascular pathology in a tightly regulated manner. Therefore, further studies are needed to explore the potential of PVAT as a target for the prevention and treatment of vascular diseases.

## Figures and Tables

**Table 1:** Anti- and proinflammatory adipocytokines secreted by PVAT and its involvement in vascular diseases.

Adipokine/cytokine	Effect on vasculature	Association with vascular diseases
Anti-inflammatory adipocytokines		
Adiponectin	(i) Vasodilator [169] (ii) ↓ adhesion molecule expression [170] (iii) ↓ oxidative stress [171]	(i) ↓ production in obesity [172] and hypertension [173]
Omentin	(i) ↓ oxidative stress [25] (ii) ↓ mitochondrial dysfunction [25] (iii) ↑ NO [174]	(i) ↓ expression in obesity [175] and CAD [176]
FGF-21	(i) ↓ oxidative stress [177] (ii) ↑ vasorelaxation [178]	(i) Improves vascular dysfunction in hypertension [179]
Vaspin	(i) ↑ insulin sensitivity [35] (ii) ↑ cytokine production [35]	(i) ↓ production in atherosclerosis [180]
Nitric oxide	(i) Vasorelaxant [167]	(i) ↓ production in atherosclerosis [181] and hypertension [182]
IL-10	(i) ↓ immune cell infiltration [37]	(i) ↓ in atherosclerosis [183]
Proinflammatory adipocytokines		
IL-6, TNF-*α*, IL-1, MCP-1	(i) ↑ immune cell infiltration [184] (ii) Endothelial dysfunction [90] (iii) ↑ ROS [185]	(i) ↑ production in obesity [184] and atherosclerosis [186, 187]
Chemerin	(i) ↑ immune cell infiltration [188] (ii) ↑ adhesion molecule expression [189]	(i) ↑ production in obesity and diabetes [190]
Leptin	(i) ↑ immune cell infiltration [54] (ii) ↑ ROS [191] (iii) endothelial dysfunction [192]	(i) ↑ production in obesity, hypertension [192], and CAD [193]
Resistin	(i) ↑ ROS [56] (ii) ↑ macrophage infiltration [56]	(i) ↑ production in atherosclerosis [194]
RANTES, Ang II	(i) ↑ immune cell infiltration [62]	(i) ↓ production in atherosclerosis [195] and hypertension [196]

Abbreviations: Ang II: angiotensin II; CAD: coronary artery disease; IL: interleukin; MCP: monocyte chemoattractant protein; NO: nitric oxide; ROS: reactive oxygen species; TNF-*α*: tumor necrosis factor-alpha.

**Table 2:** Changes related to PVAT inflammation in atherosclerosis and hypertension.

Atherosclerosis	Hypertension
(i) ↑ MCP-1 [197]	(i) ↑ IL-6, IL-17, IL-8, IL-23, IL-1*β*, TNF-*α*, TGF-*β*, MCP-1 [198–200]

(ii) ↑ RANTES [201]	(ii) ↓ adiponectin, IL-10, IL-4 [202]

(iii) ↑ IL-6, IL-1, IL-7 [203–205]	(iii) ↑ RAAS, aldosterone, and Ang II [206]

(iv) ↑ TNF-*α* [207]	(iv) ↑ activation of AT1R [208]

(v) ↓ adiponectin [209]	(v) ↑ ROS [210]

(vi) ↑ Xpr1 & Taf3 [111]	(vi) ↑ NOX [211]

(vii) ↑ PIAS1 [212]	(vii) ↑ complement C3 [213]

(viii) ↑ FFA [214]	(viii) ↑ CCR2, CCL2, CCL7, CCL8, CCL12, CCL3 [140]

(ix) ↑ ROS [215]	(ix) ↑ ET-1 [210]

(x) ↓ NO [216]	

(xi) ↓ H₂S [217]	

(xii) ↑ IFN-*γ* [218]	

(xiii) ↑ Leptin [219]	

(xiv) ↑ Visfatin [220]	

(xv) ↑ MIP-1*α* [221]	

(xvi) ↓ IgM [222]	

Abbreviations: Ang II: angiotensin II; AT1R: angiotensin II type 1 receptor; C3: component 3; CCR: CC chemokine receptor; CCL: CC chemokine ligand; ET-1: endothelin-1; FFA: free fatty acid; H_2_S: hydrogen sulfide; IFN-*γ*: interferon-*γ*; IL: interleukin; IgM: immunoglobulin M; MCP-1: monocyte chemoattractant protein-1; MIP-1*α*: macrophage inflammatory protein 1-*α*; NO: nitric oxide; NOX: NADPH oxidase; PIAS1: protein inhibitor of activated STAT1; RAAS: renin-angiotensin-aldosterone system; RANTES: regulated on activation, normal T cell expressed, and secreted; ROS: reactive oxygen species; Taf3: TATA-box binding protein-associated factor 3; TNF-*α*: tumor necrosis factor-alpha; Xpr1: xenotropic and polytropic retrovirus receptor 1.
